# Factors influencing the well-being of Asian American LGBT individuals across the lifespan: perspectives from leaders of community-based organizations

**DOI:** 10.1186/s12877-022-03590-7

**Published:** 2022-11-28

**Authors:** Alicia K. Matthews, Chien-Ching Li, Brandon Bernhardt, Shams Sohani, Xin Qi Dong

**Affiliations:** 1 Department of Population Health Nursing Science, University of Illinois at Chicago, Chicago, IL USA; 2 Department of Health Systems Management, Rush University, Chicago, IL USA; 3 The CDC Foundation, Chicago, IL USA; 4 Department of Sociomedical Sciences, Columbia University, New York City, NY USA; 5 Health Care Policy and Aging Research, Rutgers University, Institute for Health, New Brunswick, NJ USA

**Keywords:** LGBT, Asian-American, Social determinants of Health, Community-based organizations, Mental Health

## Abstract

**Background:**

Lesbian, gay, bisexual, and transgender (LGBT) individuals have documented disparities in mental health that are experienced across the life course. However, limited research has been conducted to identify the factors which contribute to evaluated risk for poor mental health among older Asian Americans who identify as LGBT. The purpose of this study was to determine the perspectives of leaders of community-based organizations about the mental health needs and concerns of their LGBT constituents from diverse Asian backgrounds.

**Methods:**

Semi-structured qualitative interviews were conducted with leaders of community-based organizations serving the needs of LGBT individuals. A qualitative framework analysis approach was used to identify, analyze and report themes within the data.

**Results:**

11 members of community organizations located in California (54.5%), Chicago (27.2%), and New York (18.1%) were interviewed. Chronic stress was identified as negatively impacting constituents’ lives and was attributed to social determinants of health, including inadequate housing, financial insecurity, discrimination, barriers to adequate health care, and immigration status. Ageism, social isolation, language barriers, and limited connections to cultural, religious, or LGBT communities were identified as factors impacting middle-aged and older adults. Participants identified homelessness, violence, and lack of parental acceptance as contributing to distress among youth and younger adults. The most vulnerable community members were identified as gender minorities, undocumented individuals, and individuals with limited English proficiencies. Organizational leaders described strategies to address social determinants.

**Conclusions:**

Asian Americans who are LGBT are confronted with substantial risks for poor mental health that are linked to modifiable social determinants of health. Organizations serving these populations play a vital role in meeting the needs of a highly underserved population.

## Background

A large body of epidemiological research has found consistent and strong associations between sexual orientation and gender identity and elevated rates of mental health conditions [1]. A systematic review of mental health among sexual minorities (lesbian, gay and bisexual individuals) has indicated that sexual minority men and women are at increased risk of having suicidal ideation and attempts, mood and anxiety disorders, and substance-related problems [1]. Similar mental health findings have been reported for gender minorities (transgender, non-binary and gender non-conforming) [2, 3]. These mental health problems are particularly critical for sexual and gender minorities who also belong to racial or ethnic minority groups [4–7] and have been observed across the life course, including among older adults [8, 9]. The factors associated with observed disparities among sexual minorities and gender minorities are complex. They are likely influenced by the same interrelated sets of social determinants of health (e.g., social, economic, and contextual factors) that drive health disparities among other underserved populations [10]. The minority stress model [11, 12] further indicates that sexual and gender minorities experience unique and chronic stressors affecting their mental health. These sources of stress include proximal stressors (e.g., internalized racism, concealment of sexual or gender identity, and perceptions of stigma) and distal stressors (e.g., the experience of prejudice, discrimination, and violence). These unique stressors experienced at the interpersonal, community, and institutional levels directly affect short- and long-term mental health outcomes [11].

Over the past decade, there have been calls from federal agencies to increase the amount, variety, and quality of research on sexual minority populations, including racial and ethnic minorities [13, 14]. According to the intersectionality theory (examining the influences of social divisions, identifications, and power relations on people’s lives, particularly for marginalized populations) [15], sexual and racial/ethnic identities are intersecting and impacting one’s ability to achieve health [16, 17]. Nevertheless, research has been scarce on mental health outcomes among sexual and gender minorities of color, who are more likely to experience discrimination and victimization [5, 18, 19]. Additional research is warranted to understand better the mental health and associated factors of sexual and gender minorities from diverse racial/ethnic communities.

Asian Americans comprise 5.4% of the total U.S. population and represent one of the fastest-growing racial or ethnic groups in the United States [20]. An estimated 325,000 or 2.8% of all Asian American adults identify as sexual minorities. Among them, about 16% are middle-aged and older Adults (40–64 years: 14%; 65+ years: 2%) [21]. In the U.S., Asian sexual minorities suffer mental health problems such as depression, anxiety, and psychological distress at higher rates than their straight Asian American counterparts [22, 23], which stem from chronic experiences of interpersonal discrimination, internalized oppression, and stigma [24–27]. This problem is especially critical among older Asian American sexual minorities who also potentially face discrimination based on age [28], ethnicity [29–31], and sexual identity [32]. Like other racial/ethnic groups, there is a great deal of cultural, language, and sociodemographic diversity among Asian Americans that may impact mental health outcomes [33, 34]. In addition, the level of acculturation, the process in which an individual adopts and adjusts to a new cultural environment, is known to influence differential mental and physical health outcomes among Asian American groups [35].

Although there are some differences based on country of origin, in general, Asian Americans report less psychological distress than non-Hispanic whites and other racial and ethnic groups [36]. However, Asian Americans are historically under-represented in sexual and gender minorities research, particularly in later adulthood. Consequently, little is known about the mental health of older Asian Americans who also identify as sexual and gender minorities. As such, the goal of the current study is to address an important gap in the mental health literature by examining the influence of sexual orientation and gender identity on the mental health of Asian Americans.

### Specific aims

Prior research conducted with social service organizations has focused on several issues related to providing aging services for LGBT adults. Findings from the extant literature have reported on the following: limited outreach to LGBT from state-level organizations providing aging services [37], factors that signal to LGBT individuals a welcoming and affirming attitude toward LGBT individuals [38] interest in LGBT cultural competency training among service providers [39], the benefits of LGBT cultural competency training for aging services providers [40], some of the issues faced by older LGBT adults [40], and predictors of utilization of aging services among midlife and older LGBT [41]. However, limited research has focused on organizations that specifically serve the needs of Asian American LGBT individuals. In partnership with LGBT (lesbian, gay, bisexual, and transgender) organizations, the purpose of this study was to understand better the mental health and well-being of the Asian American constituents served by their respective organizations and to identify risk and protective factors contributing to mental health.

## Methods

### Study design

Data for this study were conducted as part of a more extensive qualitative study of the health needs and concerns of LGBT-identified adults from various Asian American backgrounds. Data were collected from April to October 2019. Informed consent was obtained from each study participant. The Institutional Review Board of Rush University in the United States approved all research activities associated with this project (18081304-IRB01).

Participant Recruitment and Enrollment.

Staff members of LGBT organizations are in a unique position to provide input on factors that influence the health and well-being of their constituents. As such, we recruited a purposive sample of staff members of these organizations to inform our understanding of the factors that influence mental health outcomes and discuss available resources and approaches to improve outcomes in this group. Participant recruitment was conducted by searching an online directory of the LGBT Asian, Asian American, South Asian, and Pacific Islander organizations in the U.S. We identified 35 organizations providing direct services and programming to the target population. http://www.nqapia.org/wpp/wp-content/uploads/2013/06/NQAPIA-Queer-Asian-Compass-Report.pdf. In addition, we also researched other general LGBT organizations that serve older LGBT populations. Introductory letters were sent out to potential organizational leadership with requests for research participation. A total of 11 members from separate organizations agreed to participate in the study.

### Data collection

Semi-structured interviews were conducted in English by project staff over the telephone. Interviews were conducted according to standardized methodology, including trained interviewers, a focused interview guide, post-session debriefings, and careful review of verbatim transcriptions of audiotapes of each interview. During the 60 -minute telephone interviews, we explored the following topics relevant to the mental health of Asian American LGBT persons: 1). What are the major mental health concerns experienced by members of the Asian LGBT communities? 2) What do you see as some of the root causes of those issues? 3) What influence does trauma, discrimination, and minority stress have on mental health? 4) Who do you see as the most vulnerable members of the communities you serve, and what are their unique concerns? 5) What are your thoughts about the appropriate priorities and strategies to address the needs of the communities you serve, especially the most vulnerable among them? Study participants were asked to respond to interview questions based on their experiences with the populations or groups that their organization serves. As compensation, participants received a $50.00 gift card.

#### Data analysis

All interviews were audiotaped, transcribed, and analyzed according to the methods of framework analysis [42]. Framework analysis involves systematic sifting, charting, and sorting material according to identified issues and themes [43]. Transcripts were read repeatedly to identify key themes and categories, then developed into a framework for coding the body of interview data. The initial coding scheme included the above broad research categories. Additional subthemes that emerged were also be coded and summarized. These design and analytic approaches are used to obtain information about a topic (i.e., sources of trauma and minority stress) and are appropriate for applied research [42, 43].

## Results

Table 1 summarizes information about the study participants. A total of *N* = 11 key staff from community-based organizations were interviewed for the study. Seven of the 11 organizations provided services exclusively for Asian American LGBT individuals and communities. Three organizations were LGBT organizations serving racially/ethnically diverse constituents, and the final organization was an exclusively Asian serving organization. Organization staff interviewed included program directors (*n* = 6), program managers (*n* = 4) and a regional organizer (*n* = 1). Community organizations were in cities with larger populations of Asian Americans, including San Francisco (n = 6), Chicago (*n* = 3), and New York City (*n* = 2) were interviewed. Seven out of 11 participants identified as Asian American (63%), and all were LGBT identified. Per ethical guidelines, it is important to preserve the confidentiality of study participants, especially when engaging with small and potentially identifiable communities. More specific details about community organizations or interviewees’ descriptions were not provided [44].

**Table 1:** Description of Participating Organizations

Interviewee	Role at Organization	Type of Organization	Location of Organization	LGBT serving organization	Asian Serving Organization	Offer Services for Older Adults
1	Director	Social Services	San Francisco	X	X	
2	Chief Programming Officer	Community Center	Chicago	X		X
3	Regional Organizer	Religious Organization	San Francisco	X	X	
4	Assistant Director	Social Services	New York	X		X
5	Director	Social Services	Chicago	X		X
6	Program Manager	Social Services	Chicago		X	X
7	Program Manager	Community Health Center	San Francisco	X	X	
8	Program Manager	Social Services	San Francisco	X	X	
9	Director	Social Services	San Francisco	X	X	
10	Director	Social Services	San Francisco	X	X	
11	Director	Social Services	New York	X	X	

### Qualitative results

The primary focus of the study was to understand the mental health and emotional well-being of Asian Americans who are LGBT. Next, we sought to identify those factors contributing to mental health outcomes. The final objective was to articulate potential strategies to improve services for this population. Significant qualitative findings from the individual telephone interviews are described in the next section and organized based on broad categories examined during the study and the subthemes that emerged. The study results are organized under these general sections. Where appropriate, illustrative quotes are also provided.

### Mental Health

Mental health disorders are a leading cause of disability in the U.S. and globally [45]. Data suggest that the most common mental health problems, including anxiety and depression, are relatively similar across racial/ethnic groups [46]. Many of the organizational directors and staff interviewed pointed to high rates of “exhaustion,” stress, and depression among the individuals served by their organizations. Prior research suggests the under-utilization of mental health services by Asian Americans, with stigma contributing to use patterns [47]. Similarly, participants indicated that a barrier to delivering needed mental health services was related to stigma and taboo associated with mental health.

*“Honestly, ‌ ‌just‌ ‌exhausted. ‌ ‌It’s‌ ‌not‌ ‌necessarily‌ depression but‌ ‌just‌ ‌exhaustion. ‌ ‌So‌ ‌depression, ‌‌ ‌exhaustion, ‌ ‌burnt‌ ‌out. ‌ ‌It’s‌ ‌part‌ ‌of‌ ‌mental‌ ‌health. ‌”.*

*“When an individual experiences stress constantly, I think that is traumatic, and that negatively impacts mental health.”*

*“I think within the API community itself. Like it is just like we all know kind of stigmatizing or taboo to speak about mental health.”*

### Factors contributing to mental health outcomes

A range of factors was identified that impacted the mental health of constituents served by the community-based organizations included in the study. These factors can be organized according to the social determinants of health. Indeed, when asked about the factors influencing the mental health and well-being of their constituents, one participant noted, *“I would say from my experience that among API [Asian and Pacific Islanders] LGBT persons, the social determinants of health influence mental health.”* The social determinants of health are the economic and social conditions that influence individual and group differences in health status [48] and are increasingly recognized as significant contributors to health outcomes [49]. Healthy People 2020 organizes the social determinants of health around five key domains: economic stability, neighborhood, physical environment, education, community, social context, and the health care system [50]. Others have included immigration status and experiences and adverse childhood experiences as additional social determinants of health [51].

Themes associated with each of the above social determinants of health were identified by study participants as linked to poor mental health outcomes among their constituents. Each of these broad themes and related subthemes is described below.

### Immigration status and experiences

An individual’s immigration status and experiences can have profound implications for their health and well-being. As such, immigration has been identified as an essential social determinant of health [51]. Stress due to limited English proficiency and its adverse effects on employment opportunities has implications for mental health outcomes and has been widely studied. However, studies suggest that immigrants have other barriers to health, including lower levels of health insurance, less access to health care, and receipt of lower quality of care [52]. Immigrants without legal status face even more significant barriers to health, including fear of deportation, limited employment opportunities, and an inability to obtain a drivers’ license and health insurance. Although access to needed services and resources has implications for health across the life course, it becomes even more critical in providing care and services for older adults [53]. Our study participants raised many of these same issues as they discussed the needs of their Asian LGBT clients and constituents, including the relationship between immigration status and limited access to needed resources.

*“I think immigration status is a huge barrier to health. Particularly ones with language barriers. They’re isolated, to begin with, and with limited language ability … they’re also very difficult to reach.”*

*“It can be challenging to help those people, and they’re really vulnerable because some of them don’t even have the legal status.”*

*“I think the most vulnerable are immigrants who are working or lower middle class. They don’t have the social or financial resources to integrate into the larger community.”*

Immigrant stress refers to the psychological strain and distress associated with adapting to a new country. Chronic immigration-related stress has been associated with a range of poor mental health outcomes, including depression [54]. Immigration following exposure to civil unrest, natural disaster, or extreme poverty can exacerbate the everyday stressors associated with immigration [55]. One study participant pointed to the importance of pre-immigration factors on the constituents’ well-being.

*“And then also like reflecting on immigration status, like the reasons why people decide to move, uh, or immigrate to the United States are not always by choice. And we need to remember that, like, generational trauma is real.”*

Families who immigrate with young children can experience challenges associated with cultural differences related to parenting behaviors [56]. According to one current study participant, LGBT youth who belong to highly stressed immigrant families are particularly vulnerable, including being at increased risk for violence and being kicked out of their homes.

*“I’m thinking about some immigrants who are not able to cope. There’s some domestic violence inside the family, and the kids are getting hurt, and some kids are getting kicked out.”*

In addition, current immigration controversies and anti-immigration policies directed toward Latin Americans contribute to emotional problems among Asian Americans who recall the discrimination associated with anti-Asian immigration policies. The following quotes illustrate these points.

*“So, I don’t think there are necessarily like a landing pad for folks who, especially in this political climate where we are like where the message of this country is that we don’t like immigrants. I think there’s a lot of trauma and impact.”*

*“‘cause it’s what happened to us during World War II, so it’s like it’s the same thing. Like, oh, they’re different, throw them into a camp, and then try to send them back. So, I think that’s what’s the most unnerving.”*

*“‘It brings up a lot of, um, I guess intergenerational trauma because we do have, um, some people who have parents or like close family members who were in the camps, and it still kinda I guess brings about a generational trauma for some.”*

### Early childhood experiences

Adverse childhood experiences (ACES) are also considered an important social determinant of health [57]. ACES come in many forms, from physical and mental abuse to neglect and household dysfunction. ACES are strongly related to the development and prevalence of many physical and mental problems throughout a person’s lifespan [58]. Research suggests an increased risk of ACES among sexual and gender minority youth that have implications for adult outcomes. In the extant literature, adverse experiences reported by LGBTQ youth include high rates of school-based bullying and victimization, parental and family rejection, and racism [59]. Some of these experiences were noted among study participants as currently impacting the clients served by their organizations.

*“I’ve also seen trauma that’s being carried out, from parents to children, especially when parents find out their children are LGBTQ. Yeah, that trauma, especially in that early age of coming of age, stays with them for quite a while.”*

*“Some grew up in the Bay area went through high school and had been harassed repeatedly for basically being a little feminine, a little different. I can still say after knowing them for 2 or 3 years; I can still see that they are going through their journey of coming to terms with it. Uh, trying to be more comfortable with themselves.”*

*“Every single API queer person growing up doesn’t usually have a queer parent as a role model, so they all go through that lonely period. That there’s a lot of low self-esteem that built up because of that loneliness and solitude that you must endure, being who you are.”*

### Economic stability

Economic stability is a critical social determinant of health and includes poverty, employment, food security, and housing stability [50]. Researchers have found that nearly a quarter (24.6%) of LGB individuals live below the federal poverty line [60]. Consistent with this prior research, economic instability was identified by our study participants as a significant concern for the Asian LGBT community members. Financial insecurity was described as reducing access to essential needs, including participating in programming offered by their community organizations. However, it was noted that there is variability among Asian groups regarding opportunities for economic development. The loss of family safety nets was identified as a factor that increased financial vulnerability among their LGBT constituents. Study participants did not describe specific causes of financial instability. However, factors associated with economic instability among marginalized communities have been identified and include racism [61], language barriers [62], lack of legal status [63], and limited educational attainment [64]. Illustrative quotes are as follows:

*“They don’t have access - access to health care, access to housing, access to food and living. So, it takes away from one’s quality of life. Just because of their identity and added intersection of being an Asian American, they live very low-quality lives, which leads to a low, you know, life expectancy rates.”*

*“There are populations within the Asian community that does have access to economic development and others that don’t.”*

*“They lost out on their social safety net. They don’t have any family to fall back on. There’s a lack of access to finances, and basically, they just rely on themselves.*

### Housing

Housing is another social determinant of health linked to economic stability. LGBTQ individuals are more likely to experience income and housing instability across the lifespan, with LGBT youth more likely to experience homelessness. Nationally, the scarcity of beds and resources for the homeless remains a public health crisis. Further, access to affordable housing among LGBT older adults has been identified as an emerging concern. Traditionally, care for elders within many Asian cultures is cared for by children and other family members. Study participants noted that lack of children and estrangement from families of origin, including siblings, increases housing vulnerability among aging LGBT adults. Study participants described the following issues related to housing.

*“Folks who are experiencing homelessness are most vulnerable.”*

*“But I think the demand and the number of folks who are experiencing homelessness are so much greater than the resources that we can provide. Like we only have so many beds at these shelters.”*

*“You know, when it comes to LGBT older adults there, I feel like a much higher risk because so few of them have traditional, you know, conventional family structures in place.”*

*“They lost out on their social safety net almost. Because it’s like either their siblings or like their children would usually take care of you in Japanese culture.”*

A critical subtheme that emerged was securing appropriate senior housing or other assisted living facilities. Several factors were identified that were associated with senior housing accessibility due to the availability of housing units and costs of senior housing.

*“I think especially with housing like there’s so much limited housing for seniors in general, and there’s like barely any housing for like LGBTQ seniors.”*

*“very isolated older adults whether they’re LGBT or not who don’t have a social support system and who don’t have family, people for instance who need guardianship and trying to help competent guardianship assigned to them to ensure their safety and protection.*

Among individuals who can locate affordable assisted living facilities, additional issues emerge related to cultural and LGBTQ status. For example, there are limited LGBT-specific senior housing facilities; as such, many LGBT seniors will be placed in facilities that may not be trained to address the unique needs of older LGBTQ. As a result, many are faced with either hiding their sexual or gender minority status or having to *“come out again”* in an environment that may not be safe. Decisions as to whether one should disclose an LGBTQ identity represent a unique source of stress for LGBT adults who are Asian.

*“And then when you think about the staff and like who’s working in the LGBTQ senior homes like they’re not necessarily going to be familiar with your culture or your language and they’re not even necessarily going to have training on like LGBTQ cultural humility. So, I think from what I know, um, a lot of LGBTQ seniors have to like go back into the closet.”*

*“Uh, you know, at the old age having to be separated from their partner or spouse of maybe 30 years or 40 years just because of health issues or needing help with assisted living and so on. Uh, that is unique.”*

### Social and community context

The social and community context refers to the immediate physical and social settings people live or work and the cultural and institutional factors that shape them [65]. Experiences with discrimination are an essential social determinant of health linked to the social and community context. Among LGBTQ populations, experiences with discrimination are associated with disparities in mental health, physical health [66], and engagement in risky health behaviors such as tobacco use [67]. Experiences with discrimination were an overarching theme among all study participants as they described the clients and constituents they serve. Subthemes associated with discrimination were also identified and are summarized below.

### Ageism

Ageism was an issue described as impacting older LGBT adults. For example, one director noted that “Queer culture in general, it’s very youth-oriented.” Many directors and other administrative staff underscored this general sentiment and said that individuals “become invisible” as they age. Over time, older adults experience more difficulty finding community, activities to engage in, and dating opportunities. One organizational staff member noted that it is as if they become *“suspended in time, not moving forward.”* It was pointed out that many individuals have close friends who provide them with support and opportunities for socialization but that the *“larger community isn’t there to provide activities to support them.”* The following quotes capture issues for members of the LGBTQ communities as it relates to aging.

*“And all I, all I’m seeing is that ageism is an, an issue just in general, you know, LGBTQ communities. So, uh, older people, especially if it’s 50 and up, usually they become invisible.”*

*“So, you know, the biggest problem in LGBTQ communities is that we never thought we’d age. So, you know, people didn’t expect they’d get older. So that is the same, and if not more, exacerbated for LGBT Asian Americans.”*

### Gender-based discrimination

Rights and protections for gender minorities have lagged beyond those of sexual minorities. Gender minorities include individuals who identify as transgender, non-binary, or gender non-conforming (GNC). In addition, rates of transphobia remain high even within the larger LGBT communities, *“Even within the LGBTQ community, there is a stigma towards trans and GNC individuals. And that’s a challenge.”* Consequently, rates of exposure to violence and discrimination are high. For example, a report from the National Center for Transgender Equality [68] found that 63% of survey respondents reported experiencing significant levels of discrimination (i.e., verbal or physical violence, loss of job). When asked to describe the most vulnerable populations served within their agency, one organizational director replied, “*I think the most marginalized communities within the foundation are trans, GNC, trans women, and GNC youth.”* Transwomen of color were perceived to be most at risk for gender-based violence and discrimination. The literature supports this same level of violence and discrimination among Asian American Transgender women [69]. As illustrated below, this sentiment was echoed by several other organizational leaders.

*“It has been more challenging [for society] to accept the fact that some individuals are born in a body that doesn’t match their gender identity, and so the stigma is much greater for someone in the trans community.”*

*“Our trans folks, it’s like they’re a lot more susceptible to hate crimes, harassment, and violence.”*

*“It is a matter of life and death. For queer people of color, it is a matter of life and death, especially if you’re a trans woman of color.”*

“*I also think when we look at LGBT aging people of trans experience. Their safety, their comfort and accessing senior services, and their ability to be sustained in long term care facilities is of great concern.”*

### Discrimination based on race/ethnicity

Study participants stressed the importance of racism as an additional social and contextual determinant of health. They indicated that LGBT individuals who belong to racial/ethnic minority groups could experience greater exposure to discrimination. Indeed, racism within the mainstream LGBTQ community has been cited as a barrier for racial/ethnic minorities to obtain the known benefits of social connection and cohesion, impacting mental health.

*“[Name of organization] had to fight discrimination just to go into certain gay bars in the Castro area back 20 years ago. One was just back ten years ago. So, it wasn’t that long ago where there was that kind of discrimination in the gay community.”*

*“A lot of us is either we’ve faced or seen people face blatant discrimination from our cultures for being LGBT. So, a lot of us are afraid.”*

### Internalized discrimination

Internalized discrimination is a byproduct of systemic bias and oppression experienced by marginalized identity groups. Repeated exposure to negative messages about one’s social groups can cause an individual to associate oppressive messages with the core of who they are. Internalized oppression has been linked to a range of adverse physical and mental health outcomes, including depression and anxiety [70]. Study participants pointed to the role of internalized discrimination in the lives of their constituents.

*“So, there is a lot of internalized homophobia and self-hatred. Many must work through that to feel good about themselves, be out, and navigate the family situation. It’s not easy for some of the older folks.”*

*“And I think there’s trauma and internalized racism within our community. There’s like this idea of acculturation or assimilating to be westernized and adapting yourself to fit these cultural norms because of the discrimination that you face because of your skin color or the way you speak.”*

### Intersectionality

Asian Americans who also identify as LGBTQ belong to two social groups that have been historically marginalized in the United States. The intersectionality theory [71] posits that individuals’ lived and multifaceted experiences at the intersections of oppressed identities and social positions have unique experiences that must be considered.

*“Because people talk about LGBTQ stuff, you know before at least marriage equality and all of that, but I’m saying how would you even think about getting married if you are a gay person if you’re fired from a job where you’re discriminated upon because you’re API or because of your immigration status? So, the main concern there generally, I think, would be not seeing justice issues intersection.”*

*“There’s a lot of support groups for 50 plus, but what about that other intersection of, you know, 50 plus Asian American folks.”*

*“If someone is an Asian American LGBTQ individual and I’m oppressed as an LGBTQ individual and stigmatized within my Asian American community, and then I move out into the LGBT community, and I’m oppressed in there because of my racial identity too. So, the experience of stress by having that intersectionality and it means that is less possibility for support.”*

### Neighborhood and built environment

Neighborhood level factors and characteristics contribute to health and well-being [72]. The risk and protective factors associated with ethnic enclaves or densely populated ethnic neighborhoods have been shown to reduce day-to-day experiences with discrimination and bias [73]. However, research on “gay enclaves” is more mixed, with some research suggesting protective factors [74] and other reporting increases in incurred risk [75]. One study participant spoke to this reality when noting that life both inside and outside of ethnic enclaves can have a variety of impacts on the clients’ lives that they serve.

*“Um, if you’re not living in a community that like densely, um, API or a community that looks like your community, kind of like living in China town, like there are higher or lower rates of negative mental health outcomes.”*

### Education and literacy

Educational attainment and health literacy are increasingly recognized as important social determinants of health [76, 77]. The role of education on life outcomes is especially true for individuals and communities characterized by poor access to health information, English as a second language, and the digital divide. The directors described sexual health as an area in which education, health literacy, and cultural norms impact the knowledge base of the constituents served.

*“As far as LGBTQ culture is concerned, I think there’s a good amount of awareness of sexual health. But, when it comes to Asian LGBT, it’s kind of split; it depends.”*

*“As sexual health is concerned, sex itself is not spoken about, wherever the individual might come from, it’s never like part of the discussion growing up. Just a lot of taboo, and a lot of negative connotation behind it.”*

*“It just surprises the lack of information they have about HIV. It’s really basic stuff, like HIV 101, like transmission, “What is HIV?“, things like that*. *.. people don’t know that.”*

### Social support and coping

Social isolation was one of the most frequently discussed consequences of limited engagement with family members. As described by study participants, social isolation is linked to poor mental and physical health outcomes and increased vulnerability among constituents as they age. Access to social support and coping resources are strong predictors of mental health outcomes and overall quality of life. The quotes below illustrate the concerns associated with social isolation:

*“People‌ ‌‌are‌ ‌lonely. ‌ You know, folks who are self-isolated or isolated because they don’t have family or because of chronic illness and pain. If you don’t have somebody checking on you, you tend to feel bad, and your body feels worse, and you neglect your home environment, or you neglect your health.”*

*“I think as your memory starts to be impaired, it becomes an issue. Because if you aren’t connected to your family, how are you gonna function? Situations‌ ‌like‌ ‌that‌ ‌that‌ ‌I‌ ‌think‌ ‌create‌ ‌vulnerability.”*

*‌ “Well, I would say family acceptance has shown up as a significant obstacle with a Sage client. She came out as a transgender woman and was pretty much ostracized from her family of origin. We had a tough time trying to figure out where she could go and where she could live.”*

In this context, study participants also pointed to their constituents’ shared experiences of estrangement from religious institutions and cultural spaces. Below are representative quotes related to alienation from religious and cultural institutions and poor social support:

*“If you begin to believe that you’re not … you know not welcome in church, which is your support group, then issues like suicide, self-harm comes in, or people just stay away.”*

*“Many young people have an interest in learning more about the culture, but then like there’s been such a big barrier for a lot of them because usually becoming involved with the culture also means like, hiding or not talking about like their sexuality to gain access to it.”*

### Health and health care

Social determinants linked to health care include access to health care and health literacy. Lack of access to health care has been a central focus of health-related disparities because of widely divergent access levels to health care among U.S. populations. Although the Affordable Care Act has increased access to essential health care services for millions of Americans [78], barriers to services remain.

*“But a lot of people do not know how to get access to, to services, they don’t fully understand like how Medicaid works, or, understand how to get what services need.”*

Language is a crucial barrier to accessing needed health care services. Study participants noted barriers associated with limited English proficiency and health-related literacy for older LGBT adults.

*“‌I‌ ‌think‌ ‌what‌ ‌I‌ ‌see‌ ‌‌are ‌disparities‌ in communication and language accessibility.”*

*“The younger generations are fluent in medical English, but the older generation for sure is not. That causes problems with decision making and frustration with the health care system.”*

The quality of the patient-provider interaction is a component of a patient’s experience within a health care setting. Systematic bias and discrimination within the health care system have been identified as contributing to health inequalities across a range of health conditions and patient populations. Study participants described racism within the healthcare system as common among Asian LGBT identified persons with immigrant experiences. Cultural competency was also noted as a barrier to health care services.

*“Definitely racism in healthcare. The minute everybody has an accent, as a patient, you’re treated differently, in my experience, from what I’ve seen.”*

*“Cultural competency issues within healthcare that I’ve seen that have been more, more of a barrier, um, than particular disease conditions.”*

### Strategies to address root causes of mental health problems

LGBT populations are disproportionately burdened with mental health problems due to increased exposure to general and minority-specific social determinants of health. Historically, there have been multiple barriers to engagement with mental health services among Asians [79]. Stigma and lack of access to culturally appropriate mental health services have been identified as key barriers. Study participants pointed to the need for creating increased community awareness about mental health and mental health care services as a way of de-stigmatizing and normalizing mental health care.

*“And then mental health, I would say normalizing mental health or like kind of making mental health a part of regular conversation.”*

Study participants identified social determinants of health as negatively contributing to the health and well-being of Asian LGBT populations across the life course. When asked to discuss potential solutions to reduce or eliminate several of the root causes of adverse mental health outcomes, several approaches were identified that Asian serving LGBT organizations could adopt. Exposure to discrimination was viewed as pervasive. As such, anti-discrimination campaigns were viewed as being necessary for the Asian American communities. Outreach to community members by organizations was also felt to help to normalize sexual and gender minorities. Targeted areas of advocacy such as religious acceptance was also suggested as a strategy.

*“We need to be able to have open dialogues and challenge those things but also uh expose individuals who don’t have the information who have not perhaps met LGBTQ individuals to decrease the fear and negativity.”*

*“I think one of the solutions is more education, more visibility, and exposure to the LGBTQ experience. [Telling people] what it is to be LGBTQ and to normalize it. In certain circles, they continue to see LGBTQ identities as a deviant from the norm.”*

*“One of the committees I sat on is creating a national campaign, is religious acceptance.”*

Lack of parental acceptance and support has been identified as a primary risk factor for healthy development for LGBT youth. Several parent interventions effectively support parents in understanding and maintaining positive relationships with their LGBT children. A similar approach is identified as being needed for Asian American families. These interventions should be grounded in the cultural values that emphasize the importance of children. Further, it was deemed essential to have separate language-specific meetings to allow for connections among parents of LGBT youth from the same cultural and language backgrounds.

*“In Asian culture, parents love their children. We found the love for their children is so important. So, we can talk to them, connect with them, and help them understand how to love their children and accept them. It’s part of the love, and that’s, that’s how we try to connect with them too, uh, help them accept their LGBT children.”*

*“So, I think the personal connection is so important. And we also have like Vietnamese speaking parents who, uh, who, who would just provide support to other Vietnamese speaking parents or Korean speaking, Japanese speaking.”*

*“ … PFLAG having parents who are API with children who are LGBT lead workshops and facilitate groups with other parents who have children who are LGBT API and kind of help people understand like the process and like acceptance and just starting the conversations within the family within themselves.”*

Social isolation was identified as contributing to vulnerability among LGBT youth and adults. However, older adults were viewed as the most vulnerable. Consequently, several recommendations were made to increase opportunities for socialization and connection in this cohort. Some participants described the importance of online strategies for socialization. Others, however, cautioned that over-reliance on social media creates barriers for older adults, and the use of email should be continued as a means of sharing information. In addition to activities to address social isolation, study participants highlighted the importance of providing support groups to allow their constituents to discuss issues they are experiencing related to the myriad of intersectional problems confronting them. Given the relative lack of diversity in mainstream LGBT events, of importance was the creation of specific activities that allow for API LGBT communities to gather.

*“kind of normalizing, um, talking about issues that people are experiencing, being able to provide groups and like social support, um, kind of negates that.”*

*“We have to create a new space where there is a merging of social media and actual physical gatherings and how to bring that together.”*

*“When people do show up, then they realize oh it is such a new complete different experience to be seeing other fellow uh queer API together and interacting together.”*

Increasing representation and visibility of API LGBT was consistently cited as a goal. One area included expanding the involvement of API LGBTs in organizational leadership in both LGBT serving and Asian serving organizations. Extra emphasis was placed on having individuals marginalized even within the LGBT communities, including transgender or non-binary persons.

*“Folks have to be more activated to ensure that they look around the table and ensure that there’s a presentation within the Asian American, you know, um, queer and trans represented folks at the table.”*

*“I think it’s important to deliberately call out, deliberately create space for those who are being marginalized within the communities.”*

Another critical solution offered was increasing access to services and resources for the most marginalized members of the communities. These include providing free or low-cost opportunities for engagement for those individuals with limited financial resources. Further, as organizations develop programming and services, it is necessary to consider culturally informed case managers and staff who can address API clients’ language and cultural needs and sexual and gender minorities within their organizations.

*“But I think it’s important to make sure that, as an organization, you create accessibility options. If somebody cannot afford to come to your event and have no opportunity to pay $25, you need to rethink if you’re catering to that person who is working a minimum wage job and is fighting to be gay and trying to come out to his parents.”*

*“Making sure that there’s culturally competent case managers or care managers, navigators that are navigating people through the process, and spending twice as much time as they would with any other family.”*

*“For some of our services, we try to make them not feel clinical or not feel like in a doctor’s office so that uh people come and hang out here and uh and I think, and there’s not this sense of there’s something wrong with me, and therefore I have to go there. it can be very healing and can help with the normalization and de-stigmatization.”*

Each of these approaches to addressing the needs of Asian LGBT individuals by community-based organizations should be systematically evaluated by researchers and the findings used to provide practice guidelines and recommendations.

## Discussion

This study contributes to the small but growing body of research on the unique risk factors for poor mental health among LGBT Asian Americans [26, 69, 80, 81]. Directors and staff of API servicing community-based organizations provided insight into the needs and experiences of groups rarely included in LGBT research, Asian Americans. The current study results extend the prior research in this area by linking the themes that emerged from study participants to the social determinants of health. Chronic stress was identified as negatively impacting constituents’ lives and was attributed to social determinants of health, including inadequate housing, financial insecurity, discrimination, barriers to adequate health care, and immigration status. Ageism, social isolation, language barriers, and limited connections to cultural, religious, or LGBT communities identified middle-aged and older adults. Participants identified homelessness, violence, and lack of parental acceptance as contributing to distress among youth and younger adults. The present study results illustrate some of the experiences and needs of API LGBT and provide recommendations for expanding programming within existing community-based organizations that can help address many of the identified needs.

Despite the strengths, limitations of the current study should also be noted. Our study included a select sample of directors and staff members from Asian and LGBT serving organizations. However, we did not collect data directly from Asian American LGBT people receiving services. Additional research is needed to more fully understand the experiences of Asian Americans who identify as LGBT. Although small sample sizes are typical for qualitative research studies, further research is required to confirm study results. Study participants were in primarily large urban areas with high concentrations of Asian Americans. Although generalizability is not a goal of qualitative research, there may be differences in the experiences and needs of Asian Americans living in other geographical areas. To address these limitations, additional research is needed to understand better some of the cross-cutting and community-specific needs of Asian American LGBT individuals across the lifespan. Staff members from community-based organizations suggest the following areas: increasing family acceptance, reducing experiences of discrimination in larger LGBT communities, increasing access to social support, and increasing access to needed social services.

## Conclusions

Mental health disparities are well-documented within the LGBT communities. However, far less research has been conducted on the factors influencing the mental health of LGBT individuals who belong to diverse racial/ethnic groups. The study results suggest that Asian Americans who are LGBT are confronted with substantial risks for poor mental health that are linked to modifiable social determinants of health. Organizations serving these populations play a vital role in meeting the needs of a highly underserved population. Target approaches are needed to address the needs of Asian sexual and gender minorities across organizations serving LGBT communities and Asian Americans, respectively.

## Data Availability

The datasets used and/or analyzed during the current study are available from the corresponding author upon reasonable request.

## Abbreviations

LGBT: Lesbian, Gay, Bisexual, and Transgender
HIV: Human Immunodeficiency Virus.
